# XGBoost-based machine learning test improves the accuracy of hemorrhage prediction among geriatric patients with long-term administration of rivaroxaban

**DOI:** 10.1186/s12877-023-04049-z

**Published:** 2023-07-10

**Authors:** Cheng Chen, Chun Yin, Yanhu Wang, Jing Zeng, Shuili Wang, Yurong Bao, Yixuan Xu, Tongbo Liu, Jiao Fan, Xian Liu

**Affiliations:** 1 The Second Medical Center &, National Clinical Research Center for Geriatric Diseases, Chinese PLA General Hospital, Beijing, 100853 People’s Republic of China; 2 Department of Cardiovascular Medicine, the 902Nd Hospital of PLA Joint Service Support Force, Bengbu, 233015 China; 3 Department of Cardiovascular Medicine, the Second Affiliated Hospital of Army Medical University, Chongqing, 400000 China; 4 Department of Information, Medical Supplies Center, PLA General Hospital, Beijing, 100853 China; 5 Department of Pharmaceutical Sciences, Beijing Institute of Radiation Medicine, Beijing, 100850 People’s Republic of China

**Keywords:** Direct oral anticoagulants, Rivaroxaban, Hemorrhage, Risk factor analysis, XGBoost

## Abstract

**Background:**

Hemorrhage is a potential and serious adverse drug reaction, especially for geriatric patients with long-term administration of rivaroxaban. It is essential to establish an effective model for predicting bleeding events, which could improve the safety of rivaroxaban use in clinical practice.

**Methods:**

The hemorrhage information of 798 geriatric patients (over the age of 70 years) who needed long-term administration of rivaroxaban for anticoagulation therapy was constantly tracked and recorded through a well-established clinical follow-up system. Relying on the 27 collected clinical indicators of these patients, conventional logistic regression analysis, random forest and XGBoost-based machine learning approaches were applied to analyze the hemorrhagic risk factors and establish the corresponding prediction models. Furthermore, the performance of the models was tested and compared by the area under curve (AUC) of the receiver operating characteristic (ROC) curve.

**Results:**

A total of 112 patients (14.0%) had bleeding adverse events after treatment with rivaroxaban for more than 3 months. Among them, 96 patients had gastrointestinal and intracranial hemorrhage during treatment, which accounted for 83.18% of the total hemorrhagic events. The logistic regression, random forest and XGBoost models were established with AUCs of 0.679, 0.672 and 0.776, respectively. The XGBoost model showed the best predictive performance in terms of discrimination, accuracy and calibration among all the models.

**Conclusion:**

An XGBoost-based model with good discrimination and accuracy was built to predict the hemorrhage risk of rivaroxaban, which will facilitate individualized treatment for geriatric patients.

## Background

Anticoagulant drugs, including vitamin K antagonists (VAKs) and direct oral anticoagulants (DOACs), have been extensively applied in clinical practice [1–4]. Compared with VAKs, DOACs, targeting specifically at factor Xa and/or thrombin, have advantages, such as predictable pharmacokinetics and pharmacodynamics, fewer food-drug and/or drug‒drug interactions, and no requirement for routine laboratory monitoring of coagulation parameters [5–8]. Due to their noninferiority in efficacy and superiority in safety, DOACs are recommended to overcome the limitations of VAKs in an increasing number of clinical practices. Data revealed that the application of DOACs has been steadily increasing over the years [9, 10].

As the first approved factor Xa inhibitor among DOACs, rivaroxaban has been widely used in the treatment of patients with nonvalvular atrial fibrillation (NVAF) to reduce the risk of stroke and systemic embolism, in the prevention of venous thromboembolism (VTE) in adult patients undergoing elective hip or knee replacement surgery, and in prophylaxis of recurrent acute deep venous thrombosis (DVT) and pulmonary embolism (PE) [11–15]. Some studies have shown that DOACs represented by rivaroxaban exhibited significantly lower mortality and intracranial hemorrhage than VAKs. However, the risk of hemorrhage remains a concern and has a significant impact on the health of patients with long-term administration [16, 17]. Recently, several groups have focused on the hemorrhagic incidence rates of rivaroxaban [18–22]. For example, the Ichiro Sakuma group analyzed the dataset of the Expand Study to explore the bleeding risk among Japanese patients with NVAF after the use of rivaroxaban [18]. The results showed a 1.2% incidence rate of major bleeding, of which the incidence rate of intracranial hemorrhage was 0.5%. Unlike warfarin, there is a lack of monitoring methods to assess the hemorrhage risk of rivaroxaban and specific antidotes during bleeding events. Therefore, it is of great importance to establish an early-warning model to precisely predict the hemorrhage risk associated with rivaroxaban.

Geriatric patients, who usually take rivaroxaban for a long time and who also have decreased renal and liver functions, are more vulnerable to hemorrhage and have a poor prognosis [23]. In this study, we investigated the risk factors that contributed to the hemorrhagic events of geriatric patients who were over 70 years old and took rivaroxaban continuously for more than 3 months. Conventional logistic regression and eXtreme Gradient Boosting (XGBoost) were utilized to establish the hemorrhage risk prediction model, of which the XGBoost-based machine learning model showed a better prediction accuracy. This study will provide a novel strategy to guide the individualized administration of rivaroxaban for geriatric patients and thus promote protocols for the safe use of rivaroxaban in clinical practice.

## Methods

### Study population and follow-up of hemorrhage

A total of 798 geriatric patients treated with rivaroxaban for anticoagulation therapy at the Chinese PLA General Hospital from 2011 to 2021 were enrolled for the establishment of hemorrhage predictive models based on multivariate logistic regression, random forest and XGBoost. In addition, 94 geriatric patients treated with rivaroxaban for anticoagulation therapy at Chinese PLA General Hospital from 2021 to 2023 were enrolled as an external validation set for random forest and XGBoost-based model. Data were collected from the our clinical database by retrieving the outpatient, physical examination and inpatient records of these patients. The inclusion criteria were as follows: subjects aged ≥ 70 years old and who had continuous oral administration of rivaroxaban ≥ 3 months with complete clinical data. Patients were excluded if they had severe trauma or major surgery within the previous 6 months or switched to other anticoagulant drugs during the treatment period. Patients whose clinical data were incomplete or had obvious recording errors were also excluded. Finally, the cohort was established for further risk analysis.

Through a well-established clinical follow-up system (telephone follow-up, outpatient and inpatient examinations), the hemorrhage information of geriatric patients was constantly tracked and recorded. Adverse hemorrhagic events, including gastrointestinal hemorrhage, intracranial hemorrhage, ocular hemorrhage, urinary bleeding, pulmonary hemorrhage, nasal hemorrhage, gingival hemorrhage, and knee joint cavity hemorrhage, were detected and recorded by doctors and nurses through clinical observation, measuring the red blood cell counts in the patients’ urine and feces, and monitoring serum hemoglobin levels.

### Clinical data collection

According to previous studies [18, 23, 24] and the characteristics of our clinical data, this study collected a total of 27 clinical indicators for risk analysis, including basic information of patients (gender, age and BMI), medication-taking information (rivaroxaban dose and combination therapy of antiplatelet drugs), underlying diseases and previous surgical history (hypertension, diabetes, high triglyceride, high cholesterol, LDL-cholesterol abnormal, lowest hemoglobin, lowest blood platelet, coronary disease, heart failure, valvulopathy, percutaneous coronary intervention (PCI), apoplexy, hemorrhage history, coagulopathy), coagulation function index (thrombin time (TT), active partial thrombin time (APTT), international normalized ratio (INR) and D-dimer), liver function (aspartate aminotransferase (AST) and alanine aminotransferase (ALT)), and renal function (BUN and creatinine). Among them, antiplatelet drugs, rivaroxaban dose and hemorrhage information were collected since the elderly patients had begun the treatment. The coagulation function index, including TT, APTT, INR and D-dimer, was obtained from the first-time physical examination data of the patients after continuous administration for more than three months. Other clinical indicators were collected before the patients started taking rivaroxaban.

### Random forest model and XGBoost model for prediction of hemorrhage risk

Random forest and XGBoost models were developed and validated with R software (version 3.6.1). Briefly, the R package “missForest” (version 1.4) was used to impute missing values, and ggplot2 graphics (version 3.3.6) was used for data analysis. To establish a hemorrhagic risk prediction model for rivaroxaban, 798 geriatric patients were randomly divided into a training cohort and a testing cohort at a ratio of 85:15. Tenfold cross-validation was performed for hyperparameter tuning of the machine learning models.

Random forest was done as previously reported [25, 26]. To be specific, N is used to represent the number of original training sets, and M is the number of features. For each tree node, m features are randomly selected, where m should be much smaller than M. The best splitting method is calculated based on m features using the Gini coefficient. Out-of-bag (OOB) error was used to detect the generalization ability of the model. The number of decision trees constructed in this study was 500, and four variables were randomly selected on each decision tree node. The importance of each variable was subsequently measured by calculating how much reduction each variable offers when it was added to the RF model using the mean decreased accuracy and Gini. The final model estimates the importance of each predictor by checking how much the prediction error has increased. The R packages “randomforest” (version 4.6.14), “Boruta” (version 7.0.0), and “caret” (version 6.0–90) were used to develop and validate the random forest model.

The XGBoost model uses a gradient boosting framework and is also a decision tree-based ensemble method. In the XGBoost model, we ran 100 repetitions, each with a different random undersampling, of a tenfold cross-validation experiment using random parameters each time. Then, the optimal set of parameters is obtained. In this study, we used the smallest multiclass logloss. After hyperparameter optimization, we used the following parameters: eval_metric = rmse, max_depth = 6, eta = 0.11, subsample = 0.60, colsample_bytree = 0.58, min_child_weight = 2, and max_delta_step = 1. The R package “xgboost” (version 1.5.2.1) was used to develop and validate the XGBoost model. We additionally performed sensitivity analyses in which we limited the study group to patients younger than 100 years old and reported the result of sensitivity analyses separately.

### Statistical analyses

Statistical software SPSS 15.0 was utilized to perform Fisher’s exact test, chi-square tests, and univariate and multivariate logistic regression analysis. Random forest and XGBoost were conducted using R software, and a *p* value < 0.05 was defined as statistically significant.

### Ethical considerations

The study was conducted in accordance with the principles of the Declaration of Helsinki. The research proposal was approved by the Ethics Committee and the Institutional Review Committee of Chinese PLA General Hospital (Approval Number: S2022-045–01).

## Results

### Patient characteristics

**Fig. 1:**
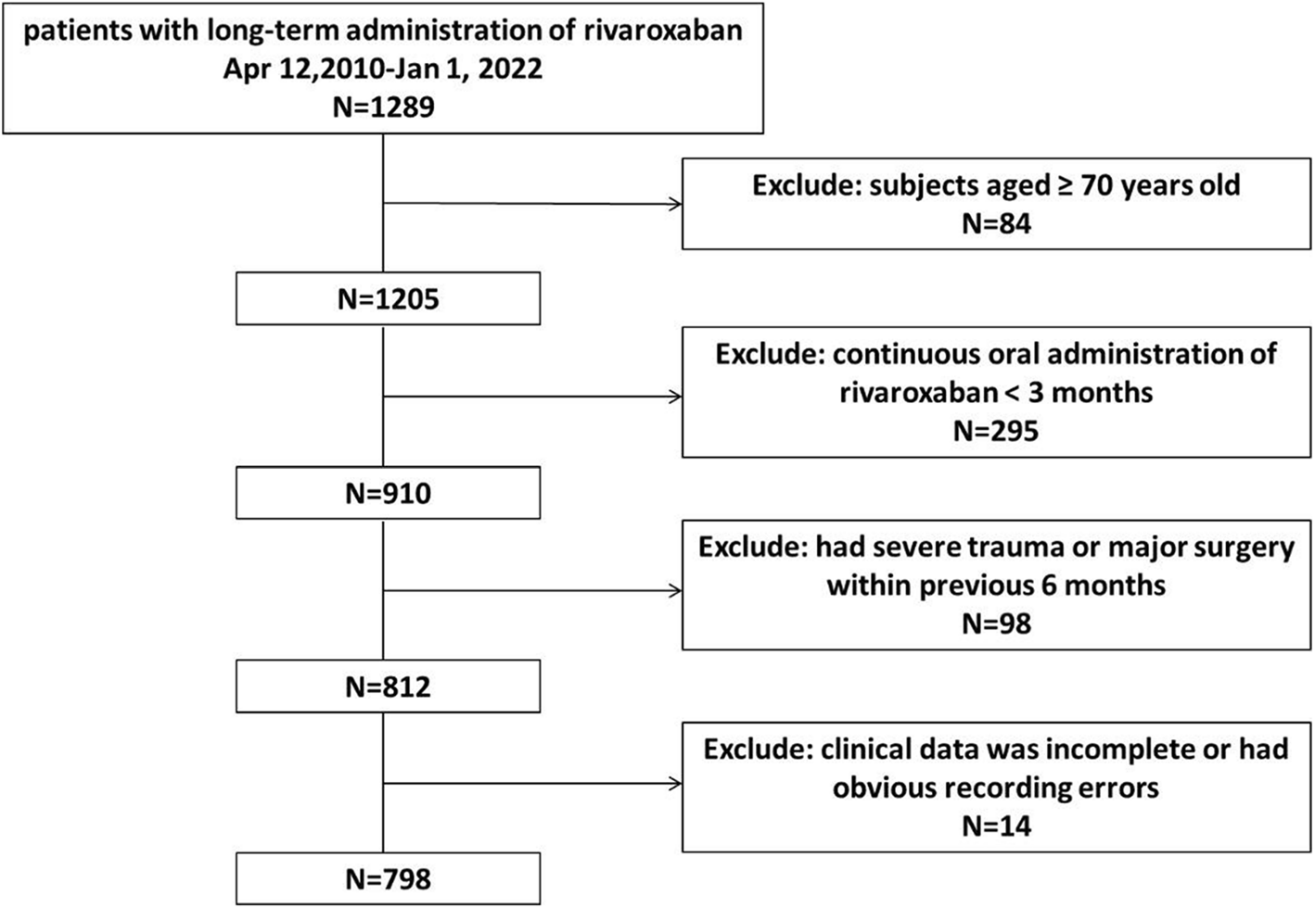
The flowchart of patient selection

A total of 1289 geriatric patients were treated with rivaroxaban for anticoagulation therapy. Moreover, 491 patients were excluded according to the exclusion criteria (Fig. 1). The baseline characteristics of the patients in the training and testing set are summarized in Table 1. Overall, this study enrolled a total of 798 geriatric patients suffering from diseases such as NVAF, VTE, PE or DVT who were treated with rivaroxaban for anticoagulation therapy. Among them, 112 patients (14.0%) had adverse hemorrhagic events during the treatment. The population distribution of 27 clinical indicators, including gender, age, BMI, rivaroxaban dose, antiplatelet drugs, hypertension, diabetes, high triglyceride, high cholesterol, LDL-cholesterol abnormal, lowest hemoglobin, lowest blood platelet, coronary disease, heart failure, valvulopathy, PCI, apoplexy, hemorrhage history, coagulopathy, TT, APTT, INR, D-dimer, AST, ALT, BUN and creatinine, is listed in Table 1.

**Table 1:** Distribution of patients’ characteristics and prognosis analysis for the establishment of predictive models

Risk factor	Total Patients, n(%) (*n* = 798)	None Hemorrhage, n(%)(*n* = 686)	Hemorrhage, n(%)(*n* = 112)	*P* Value^a^	*P* Value^b^	OR (95% CI)
Gender, n(%)						
Female	58(7.3)	52(7.6)	6(5.4)			Ref
Male	740(92.7)	634(92.4)	106(94.6)	0.555	0.403	1.45(0.61–3.46)
Age, n(%)						
< 80	116(14.5)	111(16.2)	5(4.5)			Ref
≥ 80	682(85.5)	575(83.8)	107(95.5)	**0.000**	**0.002**	**4.13(1.65–10.36)**
BMI, n(%)						
< 18.5	193(29.4)	161(28.2)	32(37.2)		0.064	Ref
18.5–23.9	275(41.9)	237(41.6)	38(44.2)		0.410	0.81(0.48–1.35)
> 23.9	188(28.6)	172(30.2)	16(18.6)	0.059	**0.020**	**0.47(0.25–0.89)**
Rivaroxaban dose, n(%)						
< 10 mg	265(33.2)	233(34.0)	32(28.6)		0.115	Ref
10 mg	477(59.8)	401(58.5)	76(67.9)		0.155	1.38(0.89–2.15)
> 10 mg	56(7.0)	52(7.6)	4(3.6)	0.107	0.294	0.56(0.19–1.65)
Antiplatelet drugs, n(%)						
no	387(48.5)	346(50.4)	41(36.6)			Ref
yes	411(51.5)	340(49.6)	71(63.4)	**0.008**	**0.007**	**1.76(1.17–2.66)**
Hypertension, n(%)						
no	163(20.4)	141(20.6)	22(19.6)			Ref
yes	635(79.6)	545(79.4)	90(80.4)	0.900	0.825	1.06(0.64–1.75)
Diabetes, n(%)						
no	452(56.6)	389(56.7)	63(56.2)			Ref
yes	346(43.4)	297(43.3)	49(43.8)	1.000	0.928	1.02(0.68–1.52)
High triglyceride, n(%)						
no	215(26.9)	188(27.4)	27(24.1)			Ref
yes	583(73.1)	498(72.6)	85(75.9)	0.493	0.466	1.19(0.75–1.89)
High cholesterol, n(%)						
no	481(60.3)	420(61.2)	61(54.5)			Ref
yes	317(39.7)	266(38.8)	51(45.5)	0.178	0.176	1.32(0.88–1.97)
LDL-cholesterol abnormal, n(%)						
no	321(40.2)	281(41.0)	40(35.7)			Ref
yes	477(59.8)	405(59.0)	72(64.3)	0.301	0.294	1.25(0.82–1.89)
Lowest_hemoglobin						
< 120	703(88.1)	593(86.4)	110(98.2)			Ref
≥ 120	95(11.9)	93(13.6)	2(1.8)	**0.000**	**0.003**	**0.12(0.03–0.48)**
Lowest_blood_platelet						
< 100	21(2.6)	18(2.6)	3(2.7)			Ref
≥ 100	777(97.4)	668(97.4)	109(97.3)	0.973	0.973	0.98(0.28–3.38)
Coronary disease, n(%)						
no	186(23.3)	170(24.8)	16(14.3)			Ref
yes	612(76.7)	516(75.2)	96(85.7)	**0.016**	**0.016**	**1.98(1.13–3.45)**
Heart failure, n(%)						
no	713(89.3)	617(89.9)	96(85.7)			Ref
yes	85(10.7)	69(10.1)	16(14.3)	0.187	0.181	1.49(0.83–2.68)
valvulopathy, n(%)						
no	750(94.0)	646(94.2)	104(92.9)			Ref
yes	48(6.0)	40(5.8)	8(7.1)	0.526	0.589	1.24(0.57–2.73)
PCI, n(%)						
no	734(92.0)	632(92.1)	102(91.1)			Ref
yes	64(8.0)	54(7.9)	10(8.9)	0.707	0.703	1.15(0.57–2.33)
apoplexy, n(%)						
no	464(58.1)	413(60.2)	51(45.5)			Ref
yes	334(41.9)	273(39.8)	61(54.5)	**0.004**	**0.004**	**1.81(1.21–2.71)**
Hemorrhage history, n(%)						
no	702(88.0)	614(89.5)	88(78.6)			Ref
yes	96(12.0)	72(10.5)	24(21.4)	**0.002**	**0.001**	**2.33(1.39–3.89)**
Coagulopathy, n(%)						
no	753(94.4)	653(95.2)	100(89.3)			Ref
yes	45(5.6)	33(4.8)	12(10.7)	**0.024**	**0.015**	**2.38 (1.19–4.75)**
TT, n(%)						
< 15	49(6.2)	42(6.1)	7(6.2)		0.377	Ref
15–21	711(89.4)	608(89.0)	103(92.0)		0.969	1.02(0.45–2.32)
> 21	35(4.4)	33(4.8)	2(1.8)	0.346	0.226	0.36(0.07–1.87)
APTT, n(%)						
< 30	22(2.8)	20(2.9)	2(1.8)		0.265	Ref
30–45	662(83.2)	573(83.8)	89(79.5)		0.557	1.55(0.36–6.76)
> 45	112(14.1)	91(13.3)	21(18.8)	0.260	0.284	2.31(0.50–10.65)
INR, n(%)						
0.8–1.2	689(86.4)	594(86.7)	95(84.8)			Ref
> 1.2	108(13.6)	91(13.3)	17(15.2)	0.554	0.588	1.17(0.67–2.05)
Ddimer, n(%)						
≤ 0.5	202(25.5)	180(26.4)	22(20.0)			Ref
> 0.5	591(74.5)	503(73.6)	88(80.0)	0.194	0.157	1.43(0.87–2.35)
ALT						
≤ 40	732(91.7)	627(91.4)	105(93.8)			Ref
> 40	66(8.3)	59(8.6)	7(6.2)	0.465	0.404	0.71(0.31–1.59)
AST						
≤ 40	751(94.1)	645(94.0)	106(94.6)			Ref
> 40	47(5.9)	41(6.0)	6(5.4)	1.000	0.796	0.89(0.37–2.15)
BUN						
≤ 7.5	485(60.8)	424(61.8)	61(54.5)			Ref
> 7.5	313(39.2)	262(38.2)	51(45.5)	0.145	0.141	1.35(0.91–2.02)
Creatinine						
≤ 110	644(80.7)	556(81.0)	88(78.6)			Ref
> 110	154(19.3)	130(19.0)	24(21.4)	0.521	0.538	1.17(0.72–1.90)

“Ref” is the abbreviation for reference. In individual risk factor, the latter supgroup compares with the “Ref” supgroup yields ORs and their 95% CIs

^a^*P* value was calculated by Fisher's exact test or Pearson Chi-Square

^b^*P* value was calculated by Logistic Regression

### Hemorrhage types of geriatric patients

**Fig. 2:**
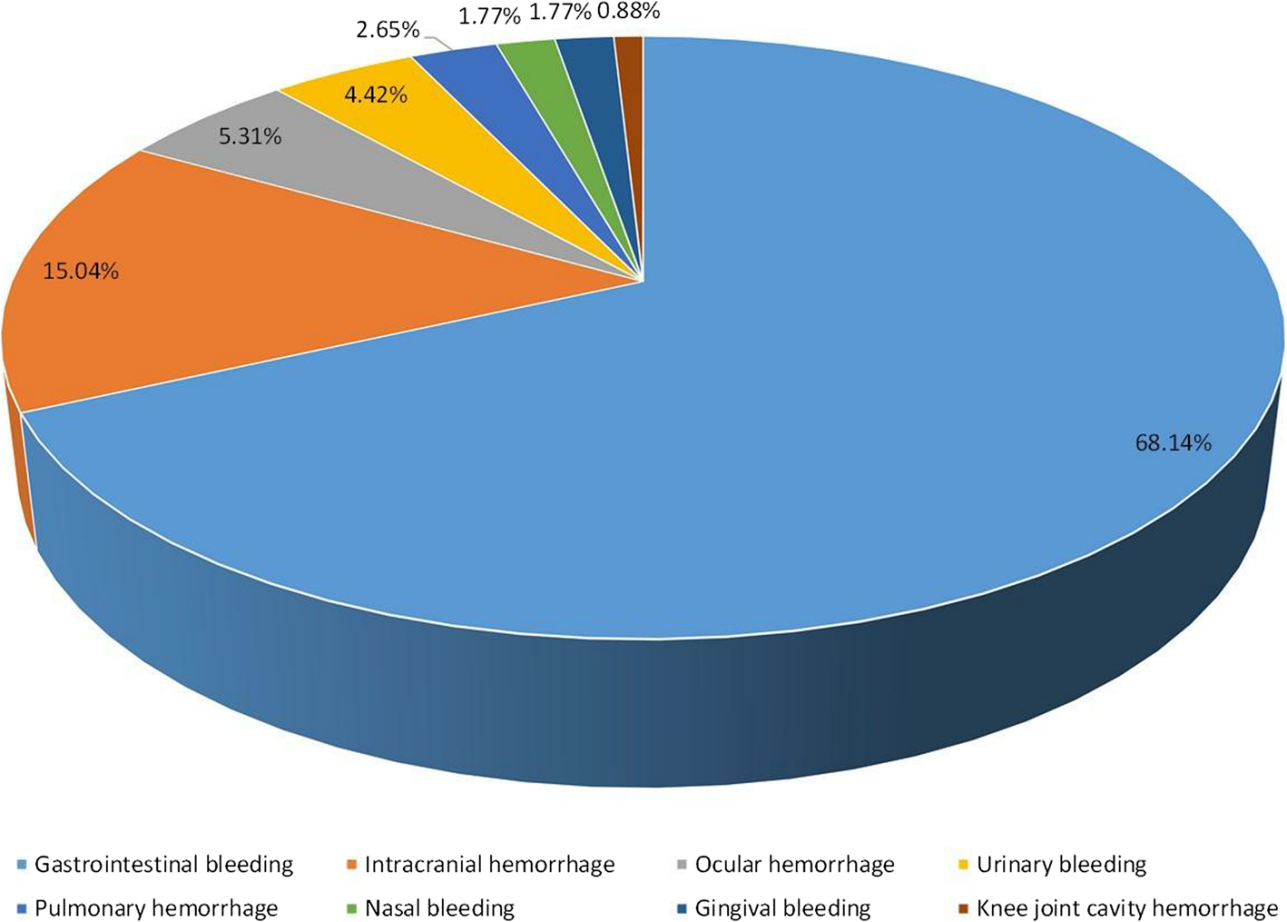
Percentage of hemorrhage types

The statistical results of the hemorrhage types are listed in Fig. 2. Among the 112 geriatric patients with hemorrhage events, 79 patients (68.14%) had gastrointestinal hemorrhage events, 17 patients (15.04%) had intracranial hemorrhage, 6 patients (5.31%) had ocular hemorrhage, 5 patients (4.42%) had urinary hemorrhage events, 3 patients (2.65%) had pulmonary hemorrhage, 2 patients (1.77%) had nasal hemorrhage events, 2 patients (1.77%) had gingival hemorrhage events, and only one patient (0.88%) had knee joint cavity hemorrhage. Gastrointestinal and intracranial hemorrhage were the most common bleeding types caused by rivaroxaban.

### Analysis of hemorrhagic risk factors and construction of a prediction model by conventional logistic regression

**Fig. 3:**
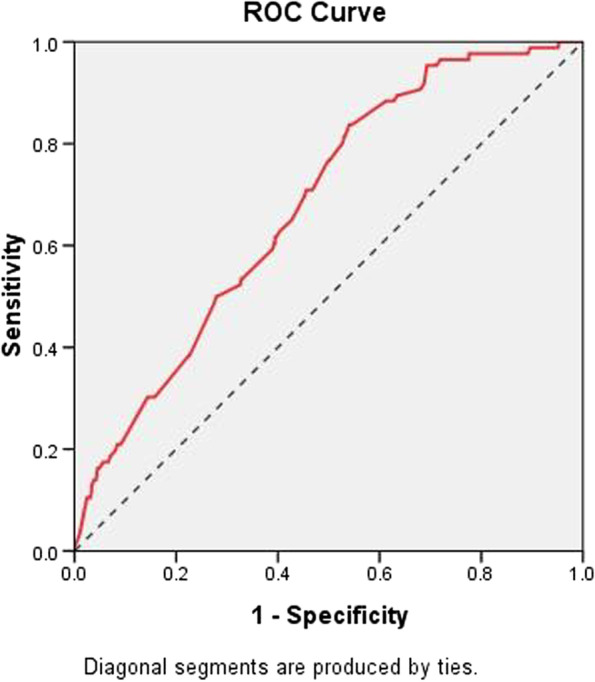
ROC curve of hemorrhage prediction model constructed by multivariate logistic regression with an AUC of 0.679

As shown in Table 1, the analysis results of the univariate logistic regression showed that risk factors including age (*P* value = 0.002, OR: 4.13, 95% CI: 1.65–10.62), BMI (*P* value = 0.020, OR: 0.47, 95% CI: 0.25–0.89), antiplatelet drugs (*P* value = 0.007, OR: 1.76, 95% CI: 1.17–2.66), lowest hemoglobin (*P* value = 0.003, OR: 0.12, 95% CI: 0.03–0.48), coronary disease (*P* value = 0.016, OR: 1.98, 95% CI: 1.13–3.45), apoplexy (*P* value = 0.004, OR: 1.81, 95% CI: 1.21–2.71), hemorrhage history (*P* value = 0.001, OR: 2.33, 95% CI: 1.39–3.89) and coagulopathy (*P* value = 0.015, OR: 2.38, 95% CI: 1.19–4.75) differed significantly between the hemorrhage and nonhemorrhage groups. Then, a multivariate logistic regression analysis was performed by incorporating the variables with significant differences obtained from the univariate logistic regression. As a consequence, the lowest hemoglobin level and hemorrhage history were identified as important risk factors in the dataset (Table 2). The hemorrhage risk of patients with the lowest hemoglobin level ≥ 120 g/L was 22% of that in patients with the lowest hemoglobin level < 120 g/L (*P* value = 0.041, 95% CI: 0.05–0.94). Meanwhile, the hemorrhage risk of patients with a history of hemorrhage was 2.04 times higher than that of patients without a history of hemorrhage (*P* value = 0.024, 95% CI: 1.10–3.78). A hemorrhage prediction model was developed by multivariate logistic regression, and the ROC curve indicated that this model had only moderate discrimination with an AUC of 0.679 (Fig. 3). To improve the accuracy of prognostication, algorithms based on machine learning were further explored to construct predictive models.

**Table 2:** Multivariate logistic regression

Risk factor	*P* Value^b^	OR (95% CI)
Age	0.166	2.01(0.75–5.38)
BMI	0.739	0.87(0.63–1.20)
Antiplatelet drugs	0.086	1.55(0.94–2.57)
Lowest_hemoglobin	**0.041**	**0.22(0.05–0.94)**
Coronary disease	0.217	1.53(0.78–299)
apoplexy	0.504	1.18(0.73–1.92)
Hemorrhage history	**0.024**	**2.04(1.10–3.78)**
Coagulopathy	0.107	1.94(0.87–4.34)

*P* value was calculated by Logistic Regression

### Analysis of hemorrhagic risk factors and construction of a prediction model based on XGBoost

**Fig. 4:**
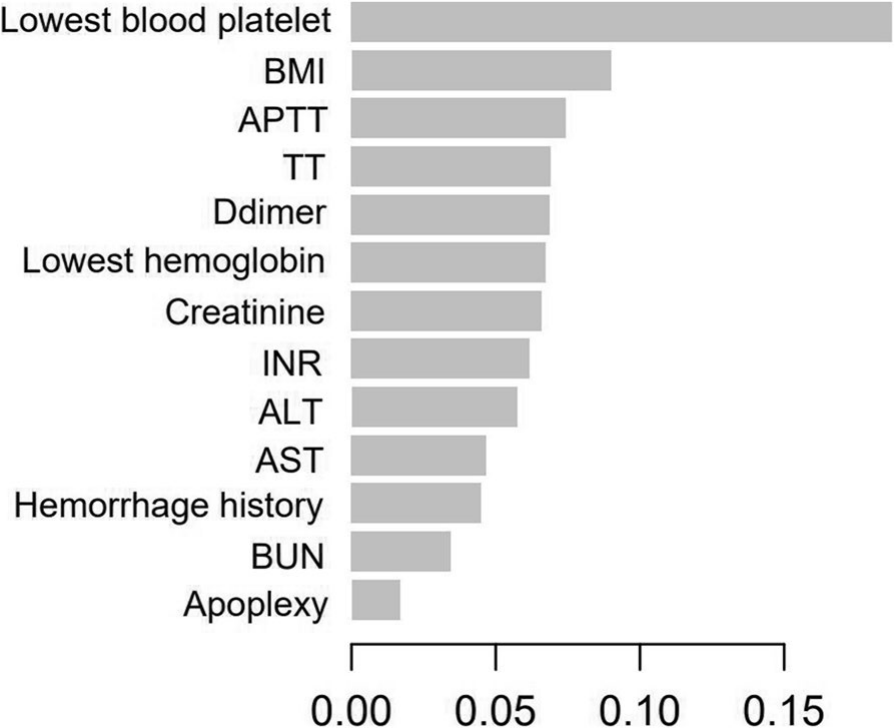
Visualize the feature importance in XGBoost model

**Fig. 5:**
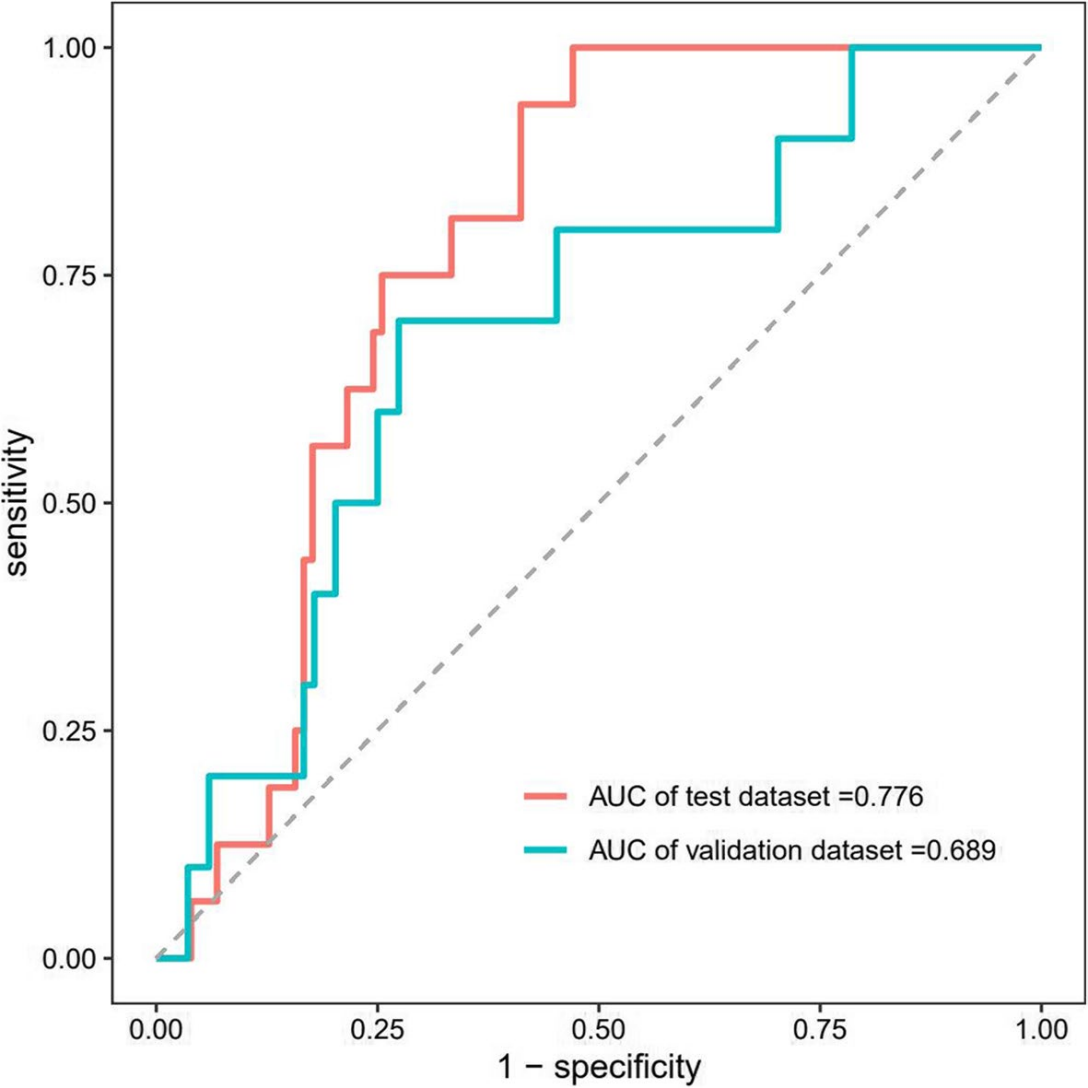
ROC for evaluating the XGBoost model’s discrimination performance in both the testing and external validation datasets. AUC in testing datasets was 0.776 and AUC in the external validation datasets was 0.689

A cohort of 94 geriatric patients with treatment of rivaroxaban were enrolled as an external validation set (Table 3). Initially, random forest was used to build the predictive model with an AUC of 0.672 for the test set and 0.610 for the external validation cohort, and this model represented poorer discriminatory power than the model built by multivariate logistic regression. XGBoost was used to establish the prediction model. First, feature selection by XGBoost identified 13 distinguished variables predisposing patients to hemorrhage: lowest blood platelet count, BMI, APTT, TT, D-dimer, lowest hemoglobin, creatinine, INR, ALT, AST, hemorrhage history, BUN and apoplexy (Fig. 4). Because it is difficult to measure BMI for bedridden geriatric patients, missing values among all the variables mainly focused on BMI features. The MissForest package was used for missing value imputation with default parameters. Then, those distinguished risk factors were utilized to develop a prediction model. In the internal validation of the testing dataset, the ROC curve showed the resulting model with an AUC of 0.776 (95% CI: 0.687, 0.864) with an accuracy, sensitivity, precision, recall, and F-1 of 0.771, 0.804, 0.921, 0.904, and 0.859, respectively (Fig. 5). In the independent external validation cohort, the model displayed decreased discrimination with an AUC of 0.689 (95% CI: 0.518, 0.860) with an accuracy, sensitivity, precision, recall, and F-1 of 0.830, 0.905, 0.905, 0.905, and 0.905, respectively (Fig. 5). With respect to sensitivity analyses, we repeated the above methodology for study groups limited to patients younger than 100 years. An analogous model training process fit models to both internal testing dataset and external validation dataset to assess the robustness of the models. The AUC of internal testing dataset was 0.784 (95% CI: 0.677–0.891) and the AUC of external validation dataset was 0.647 (95% CI: 0.451–0.843).

**Table 3:** Distribution of patients’ characteristics and prognosis analysis for external validation set

Risk factor	Total Patients, n(%)(*n* = 94)	None Hemorrhage, n(%)(*n* = 84)	Hemorrhage, n(%)(*n* = 10)	*P* Value
Gender, n(%)				
Female	8(8.5)	8(9.5)	0(0.0)	
Male	86(91.5)	76(90.5)	10(100.0)	0.593
Age, n(%)				
< 80	15(16.0)	14(16.7)	1(10.0)	
≥ 80	79(84.0)	70(83.3)	9(90.0)	0.501
BMI, n(%)				
< 18.5	22(25.3)	20(25.3)	2(25.0)	
18.5–23.9	33(37.9)	29(36.7)	4(50.0)	
> 23.9	32(36.8)	30(38.0)	2(25.0)	0.715
Rivaroxaban dose, n(%)				
< 10 mg	56(59.6)	48(57.1)	8(80.0)	
10 mg	36(38.3)	34(40.5)	2(20.0)	
> 10 mg	2(2.1)	2(2.4)	0(0)	0.368
Antiplatelet drugs, n(%)				
no	66(70.2)	59(70.2)	7(70.0)	
yes	28(29.8)	25(29.8)	3(30.0)	1.000
Hypertension, n(%)				
no	23(25.0)	23(28.0)	0(0.0)	
yes	69(75.0)	59(72.0)	10(100.0)	0.061
Diabetes, n(%)				
no	59(64.1)	53(64.6)	6(60.0)	
yes	33(35.9)	29(35.4)	4(40.0)	0.742
High triglyceride, n(%)				
no	40(42.6)	36(42.9)	4(40.0)	
yes	54(57.4)	48(57.1)	6(60.0)	1.000
High cholesterol, n(%)				
no	63(67.0)	54(64.3)	9(90.0)	
yes	31(33.0)	30(35.7)	1(10.0)	0.157
LDL-cholesterol abnormal, n(%)				
no	50(53.2)	45(53.6)	5(50.0)	
yes	44(46.8)	39(46.4)	5(50.0)	1.000
Lowest_hemoglobin				
< 120	72(76.6)	62(73.8)	10(100.0)	
≥ 120	22(23.4)	22(26.2)	0(0.0)	0.110
Lowest_blood_platelet				
< 100	3(3.2)	3(3.6)	0(0.0)	
≥ 100	91(96.8)	81(96.4)	10(100.0)	0.771
Coronary disease, n(%)				
no	28(30.4)	25(30.5)	3(30.0)	
yes	64(69.6)	57(69.5)	7(70.0)	1.000
Heart failure, n(%)				
no	72(78.3)	67(81.7)	5(50.0)	
yes	20(21.7)	15(18.3)	5(50.0)	**0.036**
valvulopathy, n(%)				
no	86(93.5)	76(92.7)	10(100.0)	
yes	6(6.5)	6(7.3)	0(0.0)	1.000
PCI, n(%)				
no	82(89.1)	72(87.8)	10(100.0)	
yes	10(10.9)	10(12.2)	0(0.0)	0.594
apoplexy, n(%)				
no	59(64.1)	54(65.9)	5(50.0)	
yes	33(35.9)	28(34.1)	5(50.0)	0.486
Hemorrhage history, n(%)				
no	82(87.2)	76(90.5)	6(60.0)	
yes	12(12.8)	8(9.5)	4(40.0)	**0.022**
Coagulopathy, n(%)				
no	92(97.9)	84(100.0)	8(80.0)	
yes	2(2.1)	0(0.0)	2(20.0)	**0.010**
TT, n(%)				
< 15	7(8.2)	7(9.2)	0(0.0)	
15–21	75(88.2)	66(86.8)	9(100.0)	
> 21	3(3.5)	3(3.9)	0(0.0)	0.511
APTT, n(%)				
< 30	2(2.4)	2(2.6)	0(0.0)	
30–45	73(85.9)	66(86.8)	7(77.8)	
> 45	10(11.8)	8(10.5)	2(22.2)	0.535
INR, n(%)				
0.8–1.2	74(87.1)	66(86.8)	8(88.9)	
> 1.2	11(12.9)	10(13.2)	1(11.1)	1.000
Ddimer, n(%)				
≤ 0.5	15(17.6)	15(19.7)	0(0.0)	
> 0.5	70(82.4)	61(82.3)	9(100.0)	0.350
ALT				
≤ 40	80(95.2)	72(96.0)	8(88.9)	
> 40	4(4.8)	3(4.0)	1(11.1)	0.370
AST				
≤ 40	79(92.9)	70(92.1)	9(100.0)	
> 40	6(7.1)	76(7.9)	0(0.0)	1.000
BUN				
≤ 7.5	48(56.5)	45(59.2)	3(33.3)	
> 7.5	37(43.5)	31(40.8)	6(66.7)	0.169
Creatinine				
≤ 110	70(82.4)	64(84.2)	6(66.7)	
> 110	15(17.6)	12(15.8)	3(33.3)	0.192

*P* value was calculated by Fisher's exact test or Pearson Chi-Square

## Discussion

Progressive increases in the incidence rates of NVAF, VTE, PE and DVT are observed with advanced age [27–30]. As previously reported, approximately one-third of all patients with AF are aged 80 or older, and the proportion of this age group will continue to rise. Moreover, the incidence of a first episode of DVT or PE in patients aged over 80 years old is estimated to be 6 times higher than that in patients less than 50 years of age. Due to the better benefit-risk profile in comparison with VAKs, rivaroxaban is recommended in the daily clinical treatment of geriatric patients for anticoagulation therapy [11–15]. However, hemorrhagic events, as one of the major side effects caused by rivaroxaban, pose a great threat to patient health. Geriatric patients showed different pathophysiologic characteristics from young patients, such as changes in the body composition of mass and muscle, impairment of liver and renal function and the presence of underlying diseases or comorbidities [27, 31]. These characteristics make geriatric patients more vulnerable to hemorrhage by affecting drug absorption, distribution, metabolism and elimination. Thus, evaluation of the potential hemorrhagic risk factors and establishment of a related predictive model are significant to guarantee the clinical safety use of rivaroxaban in geriatric patients.

In our study, we built a cohort of 798 geriatric patients with continuous treatment of rivaroxaban for more than 3 months to establish the hemorrhagic predictive model. A total of 112 geriatric patients (14.0%) had hemorrhagic events during treatment, which is higher than the 8.4% hemorrhagic rate observed in the elderly cohort (a median age of 71.66 years) reported by Hou et al. [23]. This may be attributed to the different age composition between the two cohorts, considering a median age of 92 years in our cohort, which is 20 years older than that in Hou’s cohort. According to the hemorrhagic location, all hemorrhagic events could be classified into 8 types (gastrointestinal hemorrhage, intracranial hemorrhage, ocular hemorrhage, urinary bleeding, pulmonary hemorrhage, nasal hemorrhage, gingival hemorrhage and knee joint cavity hemorrhage). Among them, gastrointestinal and intracranial hemorrhage were the most common hemorrhage types, accounting for 68.14% and 15.04%, respectively. All these results indicated that hemorrhage tendency rates could increase with age and daily clinical observation, and routine monitoring of serum hemoglobin levels and red blood cell count in the urine and feces is crucial for the safety of geriatric patients.

Candidate variables for the model were chosen from variables collected at the baseline study visit based on existing evidence and clinical relevance. Considering the influencing factors of hemorrhage reported in previous studies and some indicators closely related to bleeding according to clinical experience, 27 clinical indicators were selected in our study. Most clinical indicators, except for rivaroxaban dose and hemorrhage information, were registered before patients took rivaroxaban for the first time. Considering the possible effects of rivaroxaban dose on hemorrhage, related information from doctors’ advice in the outpatient medical record was also incorporated into predictive indicators. Hemorrhagic events occurring after the use of rivaroxaban were truthfully reported with a well-established clinical follow-up system. The frequency of telephone follow-up was once every 3 months. However, it should be noted that some potential indicators, such as the level and activity of factor Xa, might be missed. The adverse effect of factor Xa inhibitors is naturally relevant to the level and activity of factor Xa, which is rarely tested for in cardiovascular patients in most hospitals.

The univariate logistic regression revealed that age, BMI and antiplatelet drugs were significantly correlated with bleeding risk, which is consistent with previous studies of rivaroxaban and other DOACs [32–34]. Due to multiple types of clinical indicators collected in our study, lowest hemoglobin, coronary disease, apoplexy, hemorrhage history and coagulopathy were also identified as risk factors with statistical significance. The lowest hemoglobin level and hemorrhage history, which were not evaluated in previous studies, were further indicated as independent risk factors for bleeding by multivariate logistic regression. The hemorrhage prediction model constructed by multivariate logistic regression after incorporating the lowest hemoglobin level and hemorrhage history as influencing factors showed a less satisfactory AUC of 0.679.

To further optimize the prediction model, random forest was applied to build a model with an AUC of 0.672 in the internal validation of the testing dataset and 0.610 in the external validation cohort, showing a poorer discrimination than multivariate logistic regression. As an emerging machine learning algorithm based on gradient boosting, XGBoost was proposed by Tianqi Chen in 2016 and showed excellent ability to customize the loss function, normalize the regular term, and process sparse features and missing data [35, 36]. These abilities allow the model to use variables with flexibility in different areas of the output space, thus realizing automatic feature selection and the fitting of high-order interactions [37, 38]. To the best of our knowledge, the XGBoost machine learning approach has never been used before to build a bleeding risk prediction model of rivaroxaban in geriatric patients. Successfully, the hemorrhage prediction model constructed by XGBoost showed the best discrimination with an AUC of 0.776 (95% CI: 0.687, 0.864) in the internal validation of the testing dataset and 0.689 (95% CI: 0.518, 0.860) in the independent external validation cohort. Out of the 27 clinical indicators, XGBoost identified 13 distinguished variables predisposing to hemorrhage in patients, including lowest blood platelet count, BMI, APTT, TT, D-dimer, lowest hemoglobin, creatinine, INR, ALT, AST, hemorrhage history, BUN and apoplexy. Coagulation function indicators, including APTT, TT and INR, were also evaluated as risk factors by Student's t test in a previous study reported by Liang et al. [24]. The results of XGBoost model suggest that periodic monitoring of the coagulation indicators (platelet, APTT, TT, D-dimer and INR) and BMI is important to reduce the occurrence of hemorrhagic events for geriatric patients with continuous treatment of rivaroxaban. When geriatric patient's coagulation function is abnormal or their BMI is too small, doctors should pay more attention to the potentially higher incidence of bleeding events.

The XGBoost model showed a better capacity than the random forest model for predicting hemorrhage in geriatric patients treated with rivaroxaban. Random forest and XGBoost are decision tree algorithms where the training data are taken in a different manner. XGBoost is a gradient boosting-based decision tree ensemble designed to be highly efficient and scalable. Since the gradient of the data is considered for each tree, the calculation is faster and the precision is more accurate than those of random forest [39]. On the other hand, compared with random forest, XGBoost shows resistance to overfitting in datasets with imbalanced feature/outcome ratios and hyperparameters, which allows tuning for imbalanced datasets [40]. Therefore, the model built by XGBoost should be employed to predict hemorrhage risk in geriatric patients with long-term rivaroxaban treatment. We believe this XGBoost model will promote personalized medication of patients treated with rivaroxaban and contribute to its safe administration.

## Limitations

There are some limitations in our study. First, it is a single-center study. A total of 798 patients in the training and testing cohorts were from the same hospital. Moreover, considering the inclusion of multiple kinds of clinical indicators, the sample size was relatively small. These limitations may limit the interpretation of the results and lead to a wide confidence interval for the XGBoost-based bleeding prediction model. Second, clinical information, especially hemorrhagic history, was reported by the patients and susceptible to recall bias. In addition, geriatric patients over 90 years old are mostly bedridden, which resulted in missing BMI values. Third, although multivariate logistic regression analysis could adjust the influence of confounding factors to some extent, more thorough investigation of confounding factors should be conducted to further strengthen the conclusions and provide a better understanding of the underlying relationships. Above all, more solid conclusions need to be confirmed in future studies.

## Conclusions

In conclusion, 112 geriatric patients (14.0%) with long-term rivaroxaban treatment had adverse bleeding events. As the main hemorrhage types, gastrointestinal and intracranial hemorrhage accounted for 83.18% of the bleeding events in total. Three hemorrhage prediction models were constructed by applying multivariate logistic regression, random forest and XGBoost. Among them, the XGBoost model performed best with good discrimination and accuracy and identified the lowest blood platelet count, BMI, APTT, TT, and D-dimer and the lowest hemoglobin, creatinine, INR, ALT, AST, hemorrhage history, BUN and apoplexy as the most contributing features. The XGBoost predictive model will contribute to the safe clinical use of rivaroxaban for geriatric patients.

## Data Availability

The datasets used and/or analyzed during the current study are available from the corresponding author on reasonable request.

## Abbreviations

AUC: Area under curve
ROC: Receiver operating characteristic
VAKs: Vitamin K antagonists
DOACs: Direct oral anticoagulants
NVAF: Nonvalvular atrial fibrillation
VTE: Venous thromboembolism
DVT: Deep venous thrombosis
PE: Pulmonary embolism
XGBoost: EXtreme Gradient Boosting
PCI: Percutaneous coronary intervention
TT: Thrombin time
APTT: Active partial thrombin time
INR: International normalized ratio
AST: Aspartate aminotransferase
ALT: Alanine aminotransferase
